# AI-driven material discovery for energy, catalysis and sustainability

**DOI:** 10.1093/nsr/nwaf110

**Published:** 2025-03-22

**Authors:** Ning Han, Bao-Lian Su

**Affiliations:** Department of Electrical and Computer Engineering, University of Toronto, Canada; Laboratory of Inorganic Materials Chemistry (CMI), University of Namur, Belgium; State Key Laboratory of Advanced Technology for Materials Synthesis and Processing, Wuhan University of Technology, China

Artificial intelligence (AI) is revolutionizing various sectors, including science, technology, industry and daily life [1,2]. One key area where AI can make a significant impact is in material design, crucial for advancing technologies such as energy storage and catalysis [3,4]. Generative models offer an effective approach by creating novel materials tailored to specific property requirements. However, challenges remain, including low success rates in generating stable crystals and difficulties in optimizing multiple properties at once [5]. The speed at which materials are discovered directly affects the pace of technological advancements, especially in fields like carbon capture, semiconductor design and energy storage [6]. Traditionally, materials were discovered through experimentation and intuition, which limited testing possibilities and prolonged development. With advancements in high-throughput screening [7], open material databases and machine-learning-based property predictors, vast numbers of materials can now be analyzed. However, these methods are still constrained by the small pool of known materials and cannot effectively target materials with specific properties. There is growing interest in inverse design to overcome these limitations, which directly generates materials with desired properties. Techniques like generative models, evolutionary algorithms and reinforcement learning show promise, but generative models still face challenges, including producing stable materials based on density functional theory (DFT) calculations and being limited to a narrow set of elements and properties.

**Figure 1.**
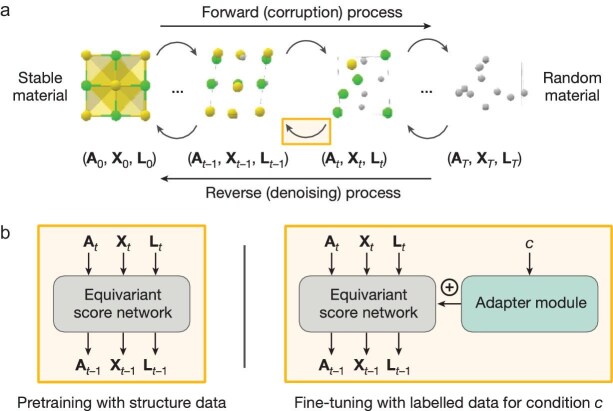
(a) MatterGen generates stable materials by reversing a corruption process through iteratively denoising a random structure. The forward diffusion process independently corrupts atom types A, coordinates X, and the lattice L towards a physically motivated distribution of random materials. (b) An equivariant score network is pre-trained on a large dataset of stable material structures to jointly denoise atom types, coordinates and the lattice. The score network is then fine-tuned with a labeled dataset through an adapter module that adapts the model using the encoded property *c*. Reprinted with permission from [8].

In a very recent *Nature* publication [8], the Microsoft Research Team introduced MatterGen, a diffusion-based generative model designed to create stable and diverse inorganic materials across the periodic table (Fig. 1a). Unlike traditional image-based diffusion models that rely on adding Gaussian noise, MatterGen employs a customized diffusion process tailored to the periodic structure and symmetries of crystalline materials. A crystalline material is characterized by its unit cell, which consists of atom types, coordinates and a periodic lattice. Each of these components undergoes a unique corruption process that respects the material's geometry, with noise distributions adjusted to ensure the stability of generated structures. To enable the design of materials with specific properties, the team introduces adapter modules that fine-tune the model using an additional dataset containing property labels (Fig. 1b). These modules are integrated into each layer of the base model, allowing it to adjust its output according to the target properties, even when the labeled dataset is smaller than the larger, unlabeled structure dataset. This fine-tuning approach is particularly valuable, as it reduces the need for extensive labeled data. The fine-tuned model is then paired with classifier-free guidance, which helps direct the material generation process toward meeting desired property constraints. MatterGen outperforms previous models by more than doubling the percentage of stable, unique and novel (SUN) materials it generates. It produces structures that are over 10 times closer to their ground-truth configurations, as verified by DFT calculations. These improvements arise from MatterGen's ability to explore a broader range of stable structures and design materials with specific properties. It generates more SUN materials in targeted systems than traditional methods and can create highly symmetric structures with desired space groups. Additionally, MatterGen can simultaneously optimize multiple property constraints, such as magnetic density and supply-chain risks. Overall, MatterGen represents a significant advancement in generative models for material discovery, offering enhanced stability and a broader range of possibilities for addressing diverse material design challenges.

The application of AI in materials design holds significant promise for advancing fields like catalysis and energy storage. MatterGen's ability to generate stable, novel materials with tailored properties can accelerate the discovery of new catalysts with optimized performance or energy-storage materials with enhanced efficiency. The model's flexibility in handling complex material constraints paves the way for the development of materials that meet the specific challenges of real-world applications, driving innovation in sustainable technologies and energy solutions.
